# Chicken *CCDC152* shares an NFYB-regulated bidirectional promoter with a *growth hormone receptor* antisense transcript and inhibits cells proliferation and migration

**DOI:** 10.18632/oncotarget.21091

**Published:** 2017-09-20

**Authors:** Shudai Lin, Wei Luo, Mingya Jiang, Wen Luo, Bahareldin Ali Abdalla, Qinghua Nie, Li Zhang, Xiquan Zhang

**Affiliations:** ^1^ Guangdong Provincial Key Lab of Agro-Animal Genomics and Molecular Breeding and Key Lab of Chicken Genetics, Breeding and Reproduction, Ministry of Agriculture, College of Animal Science of South China Agricultural University, Guangzhou 510642, P.R. China; ^2^ Agricultural College, Guangdong Ocean University, Zhanjiang 524088, P.R. China

**Keywords:** bidirectional promoter, GHR antisense transcript, coiled-coil domain containing 152, NFYB, cell cycle

## Abstract

The chicken coiled-coil domain-containing protein 152 (*CCDC152*) recently has been identified as a novel one implicated in cell cycle regulation, cellular proliferation and migration by us. Here we demonstrate that *CCDC152* is oriented in a head-to-head configuration with the antisense transcript of growth hormone receptor (*GHR*) gene. Through serial luciferase reporter assays, we firstly identified a minimal 102 bp intergenic region as a core bidirectional promoter to drive basal transcription in divergent orientations. And site mutation and transient transfected assays showed that nuclear transcription factor Y subunit beta (NFYB) could bind to the CCAAT box and directly transactivate this bidirectional promoter. SiRNA-mediated NFYB depletion could significantly down-regulate the expression of both *GHR-AS-I6* and *CCDC152*. Additionally, the expression of *GHR-AS-I6* was significantly up-regulated after *CCDC152* overexpression. Overexpression of *CCDC152* remarkably reduced cell proliferation and migration through JAK2/STAT signaling pathway. Thus, the *GHR-AS-I6–CCDC152* bidirectional transcription unit, as a novel direct target of NFYB, is possibly essential for the accelerated proliferation and motility of different cells.

## INTRODUCTION

Most genes in prokaryotes are economically organized at a high density and arranged in operons, ensuring effective co-regulation of genes [1]. In contrast, in higher eukaryotes, genes are organized at low density and arranged individually, which facilitates flexible and precise regulation [2]. Surprisingly, the sequencing data of the human genome found that more than 10% of genes are shown in a divergent or “head-to-head”configuration with an intervening sequence of less than 1 kb [3–5]. Strikingly, transcription of such gene pairs is driven by unique, bidirectional promoters that initiate transcription in both directions and contain shared elements that regulate both genes [6, 7].

Expression of gene pairs transcribed by a bidirectional promoter is likely to be correlated; the two genes always share a similar function and are regulated by the same transcription factor [8–11]. Increasing studies strongly demonstrated that these gene pairs play important roles in biological processes, such as DNA damage signaling pathway and the development of cells [12, 13], and even various diseases such as cancer [6, 14, 15]. In the human genome, no more than 20 of these gene pairs have been identified and characterized in details, including *BRCA1–NBR2*, *Mrps12–Sarsm*, and *PRR11–SKA2* [13]. However, in the chicken genome, just two gene pairs have been reported in detail, including *GPAT-AIRCR* [16, 17] and *Rap1-KARS* [18]. Previously, we have identified a novel chicken gene product, growth hormone receptor (*GHR*) antisense transcript intron 6 (*GHR-AS-I6*), a novel natural transcript initiating from the 3’ flanking region and ending in intron 6 in the opposite strand of *GHR* gene, which is partially complimentary to *GHR* messenger RNA (mRNA) and recruits to the *GHR* promoter to regulate the expression level of *GHR* mRNA [19].

The coiled-coil domain-containing (CCDC) proteins exhibit different kinds of functions due to their highly versatile folding motif [20, 21]. The coiled-coil motif is found in many proteins, such as skeletal and motor proteins, and is involved in molecular recognition systems and protein refolding [20–22]. It was also reported that CCDC proteins were involved in the process of gene transcription events, cell cycle regulation, apoptosis, and the invasion and metastasis of malignant tumor cells [23–30]. Notably, *CCDC80* (also known as Equarin) is involved in chicken eye formation [31] and is strongly expressed in the notochord of zebrafish [32]. However, the functions of numerous *CCDC* genes, such as *CCDC152*, remain unclear.

Intriguingly, we noticed that the chicken *GHR-AS-I6* and *CCDC152* are closely located on chromosome Z in a head-to-head orientation, suggesting that both genes are coordinately regulated by a unique bidirectional promoter. Here, we propose that the transcription of both *GHR-AS-I6* and *CCDC152* were controlled by a bidirectional promoter, and *CCDC152* plays role in cell cycle progression and cell migration *in vitro*.

## RESULTS

### The organization of GHR-AS-I6 and CCDC152 transcripts

**Figure 1:**
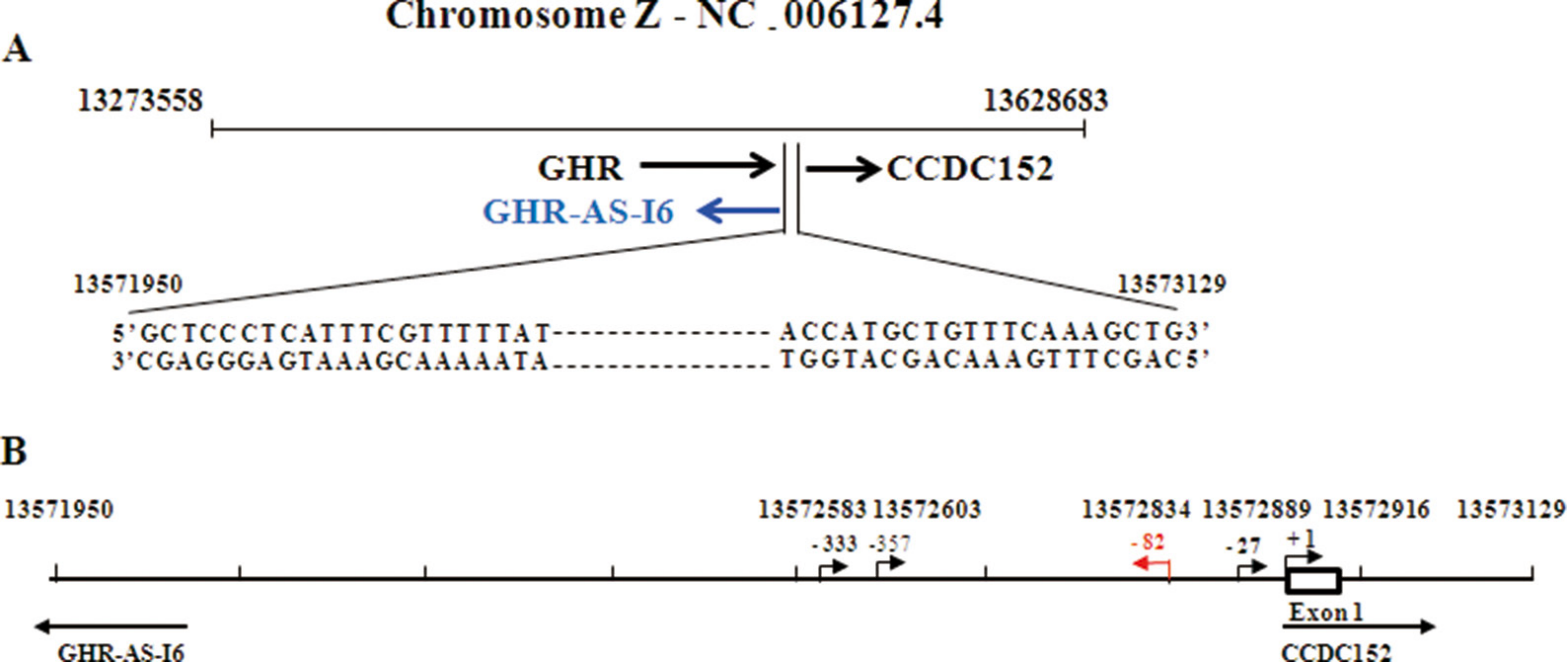
Genomic organization of the GHR-AS-I6 and CCDC152 (**A**) The location of the intervening region of *GHR-AS-I6-CCDC152* in the chicken genome. (**B**) The potential transcript start sites of *GHR-AS-I6-CCDC152* bidirectional promoter predicted by online software Fruitfly and PromoterScan. The 13572916 (+1) site was verified by 5′-RACE.

To determine whether chicken *GHR-AS-I6* and *CCDC152* are bidirectional transcripts, we used *GHR-AS-I6* [19] and the downloaded *CCDC152* sequence and employed the NCBI BLAST (http://blast.ncbi.nlm.nih.gov/Blast.cgi) to analyze the genomic organization. As shown in Figure 1A, *GHR-AS-I6* and *CCDC152* are matched to chromosome Z in a head-to-head orientation, with the transcripts separated by approximately 1000 bp. Notably, the intergenic region between *GHR-AS-I6* and *CCDC152* was 1180 bp (chrZ: 13571950 to 13573129), which was considered as an active promoter region (Figure 1A) by using network tools Promoterscan and Softberry for prediction. Taken together, the above results strongly suggest that chicken *GHR-AS-I6* and *CCDC152* are a classic head-to-head transcriptional gene pair probably driven by a unique bidirectional promoter.

### The transcription start sites of GHR-AS-I6 and CCDC152

To see the transcription start sites of *GHR-AS-I6* and *CCDC152* and to clarify the intergenic region, we first analyzed *CCDC152* using the online tools Jaspar and PROMO to predict the TATA box and transcriptional factor binding sites in the 1180 bp promoter region, respectively. We found that there was only one transcription start site (TSS) located at 13572834 for *GHR-AS-I6* and 4 TSSs located at position 13572583, 13572603, 13572889 and 13572916 for *CCDC152*, indicating that there may be various 5′ un-translated regions for *CCDC152* mRNA transcripts (Figure 1B).

To further verify the predicted results, we conducted 5′-RACE analysis using total RNAs from WRR chicken liver and leg muscle tissues. As shown in Supplementary Figure 1, different PCR products were amplified from WRR chicken liver and leg muscle tissues. Subsequent cloning and sequencing analysis of the 5′-RACE products revealed that a single major transcription start site for *CCDC152* is present at position 13572916 in chicken liver tissue (Figure 1B).

Combining the predicted analysis and our 5′-RACE results, we assigned position 13572916 as the major TSS for *CCDC152*, but we could not amplify the 5′ end of *GHR-AS-I6*. Hence, for further analysis, we designated the major transcription start site of *CCDC152* at position 13572916 as +1.

### Identification of the GHR-AS-I6–CCDC152 bidirectional promoter region

**Figure 2:**
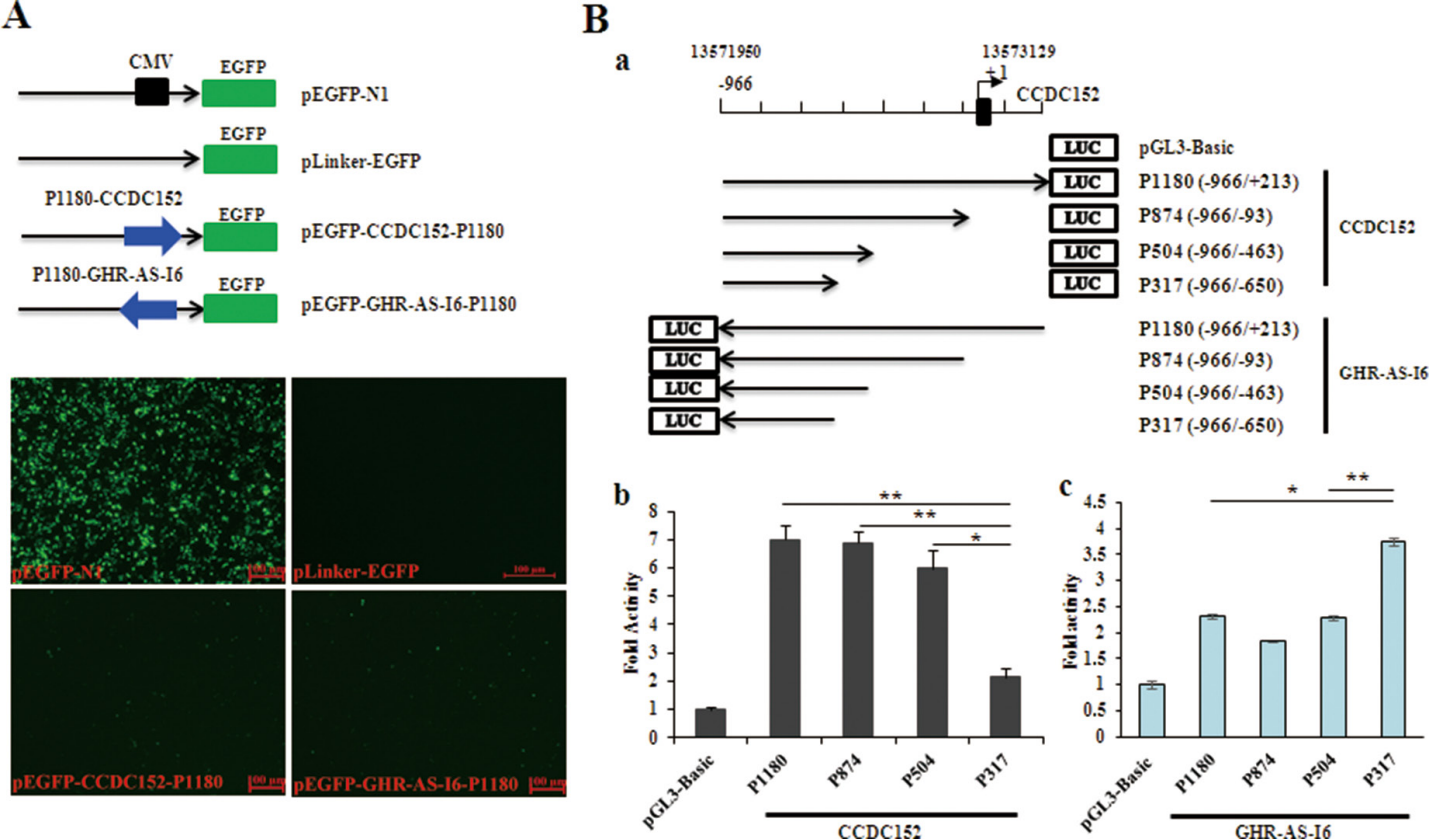
Identification of the proximal GHR-AS-I6-CCDC152 bidirectional promoter region (**A**) The green fluorescent protein (GFP) detection of both *CCDC152* and *GHR-AS-I6* directional promoter region in LMH cells transfected with positive control pEGFP-N1, and negative control pLinker-EGFP (without CMV promoter) (40 × magnification). (**B**) The upper panel shows a schematic diagram of the luciferase reporter constructs containing the indicated genomic fragments of this *GHR-AS-I6-CCDC152* pair. Positions relative to the major transcriptional initiation site of *CCDC152* (+1) are indicated. LMH cells were transiently co-transfected, in triplicate, in 24-well culture plates with the indicated luciferase reporter constructs together with the Renilla luciferase reporter plasmid (pRL-TK). Firefly and Renilla luciferase activities were measured 48 h after transfection. Data are shown as fold induction compared with the activity of cells transfected with the empty promoter-less pGL3-basic luciferase reporter vector alone. The results are shown as the mean ± S.E.M of triplicate experiments. Significance: *, ** indicate *P* < 0.05 and *P* < 0.01, respectively.

To investigate whether the intergenic region of *GHR-AS-I6–CCDC152* acts as a bidirectional promoter, a series of luciferase reporter constructs were generated by inserting various intergenic fragments of the *GHR-AS-I6–CCDC152* transcription pair in both orientations into the promoter-less pGL3-basic vector (Figure 2). The promoter activities were assessed following transient transfection of these reporters into LMH cells. As shown in Figure 2B, all deletion constructs of *CCDC152* displayed significant promoter activities with different efficiencies. Compared with pGL3-basic vector, all deletion constructs of *GHR-AS-I6* showed higher but not-significant promoter activities.

Notably, a short segment of 504 bp (–996 bp to –463 bp) was activated in both orientations in LMH cells, strongly suggesting that this genomic region displays strong constitutive bidirectional promoter activity that drives transcription of both *GHR-AS-I6* and *CCDC152*. Additionally, strong enhancers for *CCDC152* were predicted to reside in the sequence between positions –650 bp and –463 bp. Indeed, compared with GHR-AS-I6-P1180 (*P* < 0.05), GHR-AS-I6-P874 and GHR-AS-I6-P504 (*P* < 0.01), the *GHR-AS-I6* constructs P317, which exclude this fragment, displayed the highest activity in the *GHR-AS-I6* orientation. Strong silencers for *GHR-AS-I6* were predicted to reside in the sequence between positions –650 and –463. Consistent with this prediction, we observed a sharp increase in the promoter activity of GHR-AS-I6–P317 compared with that of GHR-AS-I6–P504.

**Figure 3:**
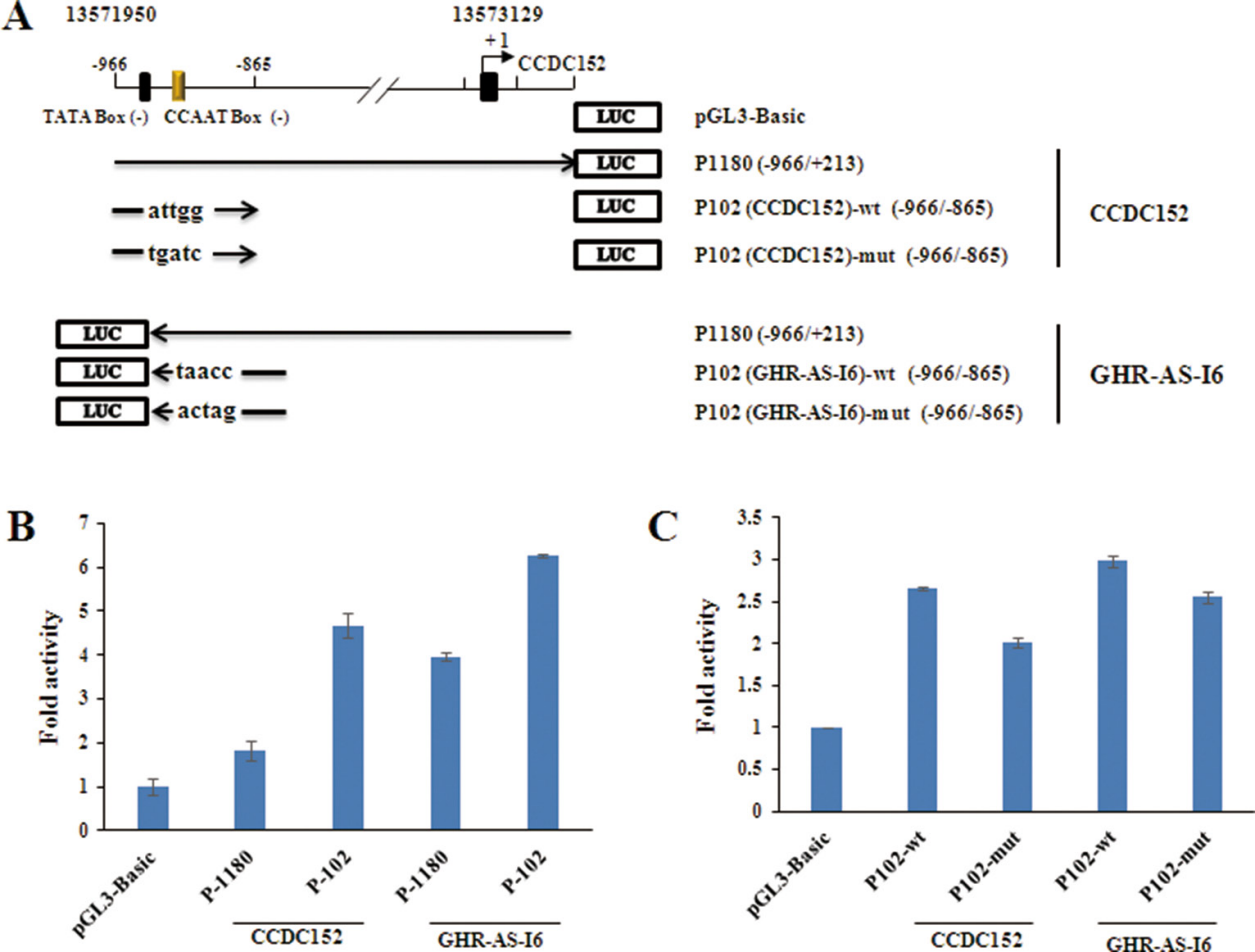
Identification of the core GHR-AS-I6-CCDC152 bidirectional promoter region (**A**) The constructs of wild type (wt) and mutation core promoter region (mut) from -966 to -865 (P102). The position relative to the major transcriptional initiation site of *CCDC152* (+1) are indicated. (**B**, **C**) Results of the reporter assay: LMH cells were transiently co-transfected with the indicated luciferase reporter constructs along with pRL-TK. Firefly and Renilla lusiferase activities were measured and analyzed 48 h after transfection, as described in Figure 2. The results are shown as the mean ± S.E.M of triplicate experiments. Significance: *, ** indicate *P* < 0.05 and *P* < 0.01, respectively.

To further minimize the genomic region of the *GHR-AS-I6–CCDC152* bidirectional promoter, additional luciferase reporter constructs containing the indicated 13571950 to 13572051 (102 bp) were generated and transiently transfected into LMH cells (Figure 3). The luciferase assay revealed that all the constructs including the shortest one, P102 (–996/–895), exhibited remarkable promoter activities, suggesting that a minimal (core) *GHR-AS-I6–CCDC152* bidirectional promoter is present within the genomic region between *–*996 and *–*895 (Figure 3). These findings strongly suggested that *GHR-AS-I6* and *CCDC152* transcripts are classic head-to-head transcriptional pair, and possibly driven by a bidirectional promoter.

### The NFYB binding sites in the bidirectional promoter

**Figure 4:**
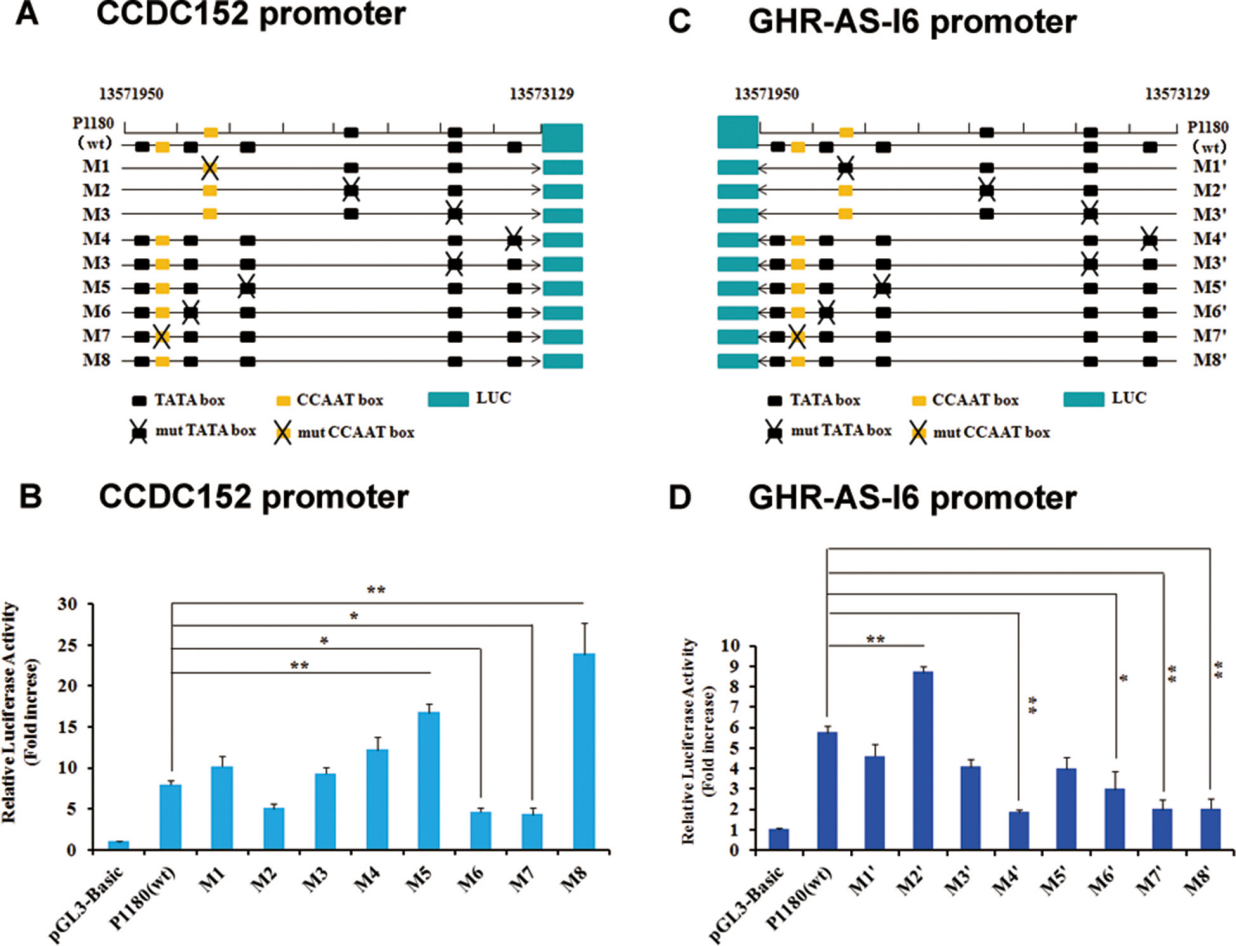
Identification of the NFYB binding sites of the bidirectional promoter The potential TATA box and CCAAT box in the bidirectional promoter and the schematic structure of a serial mutation constructs of TATA box and CCAAT box (**A, C**). The promoter activity of the normal and mutation bidirectional promoter in LMH cells (**B, D**). Significance: *, ** indicate *P* < 0.05 and *P* < 0.01, respectively.

We used online tools Jaspar and PROMO to identify putative consensus binding sequences, predominantly the CCAAT box for NFYB, within the *GHR-AS-I6–CCDC152* bidirectional promoter region (Figure 4A and 4C). To confirm whether the NFYB influences the transcriptional activity of this bidirectional promoter, we conducted site mutation constructs and transient transfection assays. For *CCDC152*, promoter mutated TATA box (Figure 4A and 4B, M2, M6) and CCAAT box (Figure 4A and 4B, M7) decreased sharply in activity, while the mutation in M5 and M8 sites resulted in a different increase in its promoter activity (Figure 4B). For *GHR-AS-I6* promoter, mutations of TATA box and CCAAT box decreased promoter activity in the excluded M2′ site, especially mutations in M4, M6, M7 and M8 sites (Figure 4C and 4D). Considering Figure 3C, the CCAAT box plays an important role in maintaining bidirectional promoter activity. Taken together, the TATA box in M6 site and the CCAAT box in M7 site play very important roles in activating the bidirectional promoter. Thus, we next focused on M7, the NFYB binding site, and verified its role in the transcriptional regulation of the *GHR-AS-I6–CCDC152* bidirectional promoter.

### NFYB combines with NFYC to transactivate transcription of the bidirectional promoter

**Figure 5:**
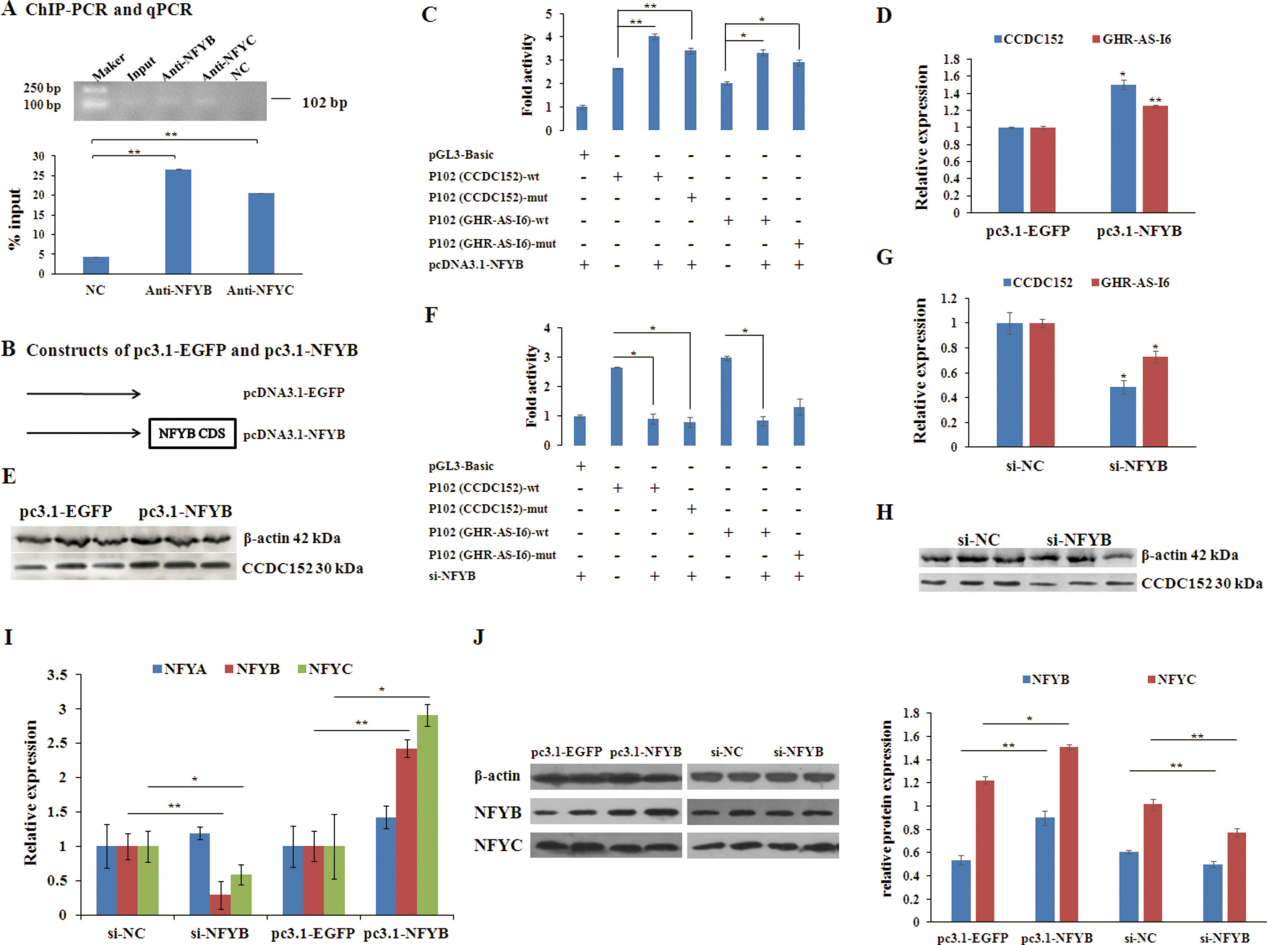
NFYB directly transactivates the transcription of both GHR-AS-I6 and CCDC152 (**A**) ChIP assay was used to detect the binding of NFYB and NFYC proteins to CCAAT box of the bidirectional promoter. (**B**) The constructs of overexpression of *NFYB* (pc3.1-NFYB) and control cells transfected with the empty vector (pc3.1-EGFP). Relative expression levels of *CCDC152*, *GHR-AS-I6* and NFYA/B/C are shown after overexpressing *NFYB* and interfering *NFYB* (**C, F, I**). The bidirectional promoter activity has been changed after inducing *NFYB* (**D**) and interfering *NFYB* (**G**). The protein level of CCDC152 and NFYB were up-regulated and down-regulated after overexpressing *NFYB* and interfering *NFYB* (**E, H, J**), respectively. Significance: *, ** indicate *P* < 0.05 and *P* < 0.01, respectively.

Numerous studies have demonstrated that the transcription factor NFYB plays a fundamental role in the transcriptional regulation of cell cycle genes, particularly G2/M genes [33–35]. From the ChIP assay (Figure 5A), we confirmed that the NFYB/C could bind to the CCAAT box. And to give insight into whether NFYB affects the expression of *GHR-AS-I6* and *CCDC152*, LMH cells were transiently transfected with pc3.1-NFYB overexpression or interference with *NFYB* (si-NFYB) (Figure 5B), or respective controls. When *NFYB* overexpressed, the expression levels of both *GHR-AS-I6* and *CCDC152* were significantly increased (Figure 5C).

Next, to understand whether NFYB activates transcription of *GHR-AS-I6–CCDC152* bidirectional promoter, LMH cells were co-transfected with an *NFYB* expression vector (pc3.1-NFYB) and/or a series of *GHR-AS-I6–CCDC152* luciferase reporters (Figure 3A, Figure 5B and 5D). The luciferase reporter assay revealed that overexpression of *NFYB* caused a significant increase in luciferase activities of the shortest constructs GHR-AS-I6-P102 and CCDC152-P102, strongly suggesting that NFYB activates the *GHR-AS-I6–CCDC152* bidirectional promoter in both orientations (Figure 5D). Conversely, knockdown of *NFYB* caused a significant decrease in the luciferase activity of the shortest constructs GHR-AS-I6-P102 and CCDC152-P102 (Figure 5F and 5G).

Furthermore, we performed qRT-PCR and Western blot to detect whether NFYB was involved in regulating the expression of CCDC152 protein and how it works. The results showed that the expression levels of CCDC152 and NFYC were increased by the overexpression of *NFYB* (Figure 5E, 5I and 5J); conversely, their expression decreased with knockdown of *NFYB* (Figure 5H, 5I and 5J). Taken together, NFYB could react with NFYC but not NFYA to transactivate the bidirectional promoter to launch *GHR-AS-I6* and *CCDC152* activity.

### CCDC152 regulates the transcription of GHR-AS-I6 and itself

**Figure 6:**
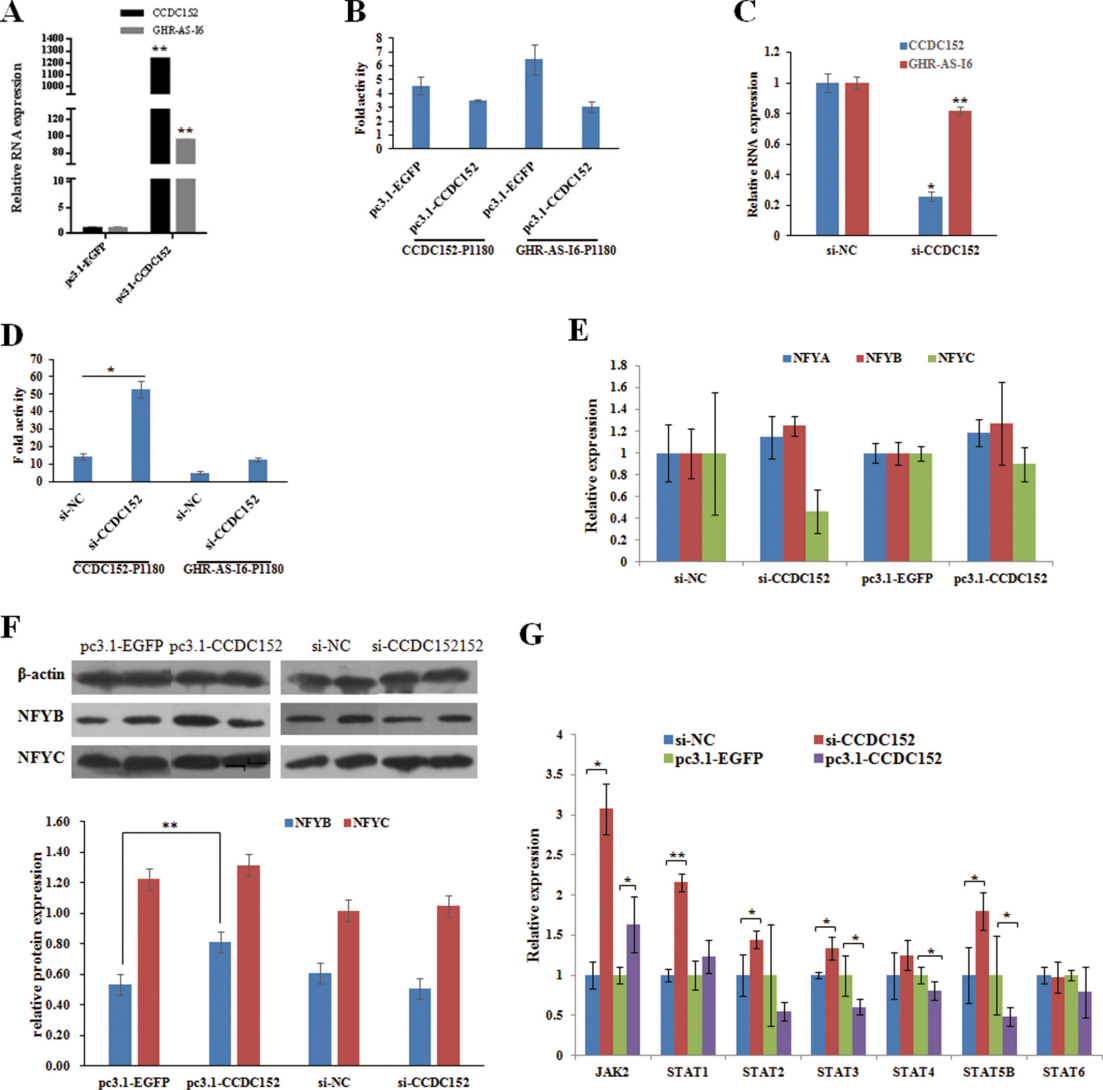
CCDC152 down-regulates the transcriptional activity of GHR-AS-I6-CCDC152 bidirectional promoter in LMH cells The relative expression levels of *GHR-AS-I6* (**A, C**), NFYA-C, JAK2 and STATs (**E–G**), and the bidirectional promoter activity (**B, D**) were changed by overexpressing and interfering *CCDC152*. Significance: *, ** indicate *P* < 0.05 and *P* < 0.01, respectively.

Here, we asked whether the CCDC152 could influence the expression of *GHR-AS-I6* and *CCDC152.* It has been reported that CCDC proteins can play various roles due to their highly versatile folding motif [20]. *CCDC* genes take part in the processes of gene transcription, apoptosis and the cell cycle and even in the invasion and metastasis of malignant tumor cells [14, 29]. As expected, from the result of overexpression of *CCDC152* in LMH cells and qRT-PCR, we found that the expression of *GHR-AS-I6* is significantly increased (Figure 6A). However, the promoter activities in both orientations are decreased (Figure 6B). When interfering with *CCDC152*, the expression of *GHR-AS-I6* was significantly decreased (Figure 6C), while promoter activities in both orientations are increased (Figure 6D).

To investigate how the CCDC152 affect the expression of *GHR-AS-I6* and *CCDC152*, we first performed qRT-PCR assay and Western blot of NFYB and NFYC. The result showed that the expression of both *NFYB* and *NFYC* didn't change with changing *CCDC152* expression (Figure 6E), but the NFYB protein was significantly increased by overexpressing *CCDC152* (Figure 6F). In addition, we also analyzed the expression of genes in JAK/STAT pathway. The expressions of *JAK2*, *STAT1-3* and *STAT5B* were increased with knockdown of *CCDC152*, and the *STAT3, STAT4* and *STAT5B* was decreased with significance by knockdown of *CCDC152* (Figure 6G). These observations support the hypothesis that CCDC152 functions as a transcriptional inhibitor of *GHR-AS-I6-CCDC152* bidirectional promoter in LMH cells.

### Silencing of CCDC152 increases cell proliferation

**Figure 7:**
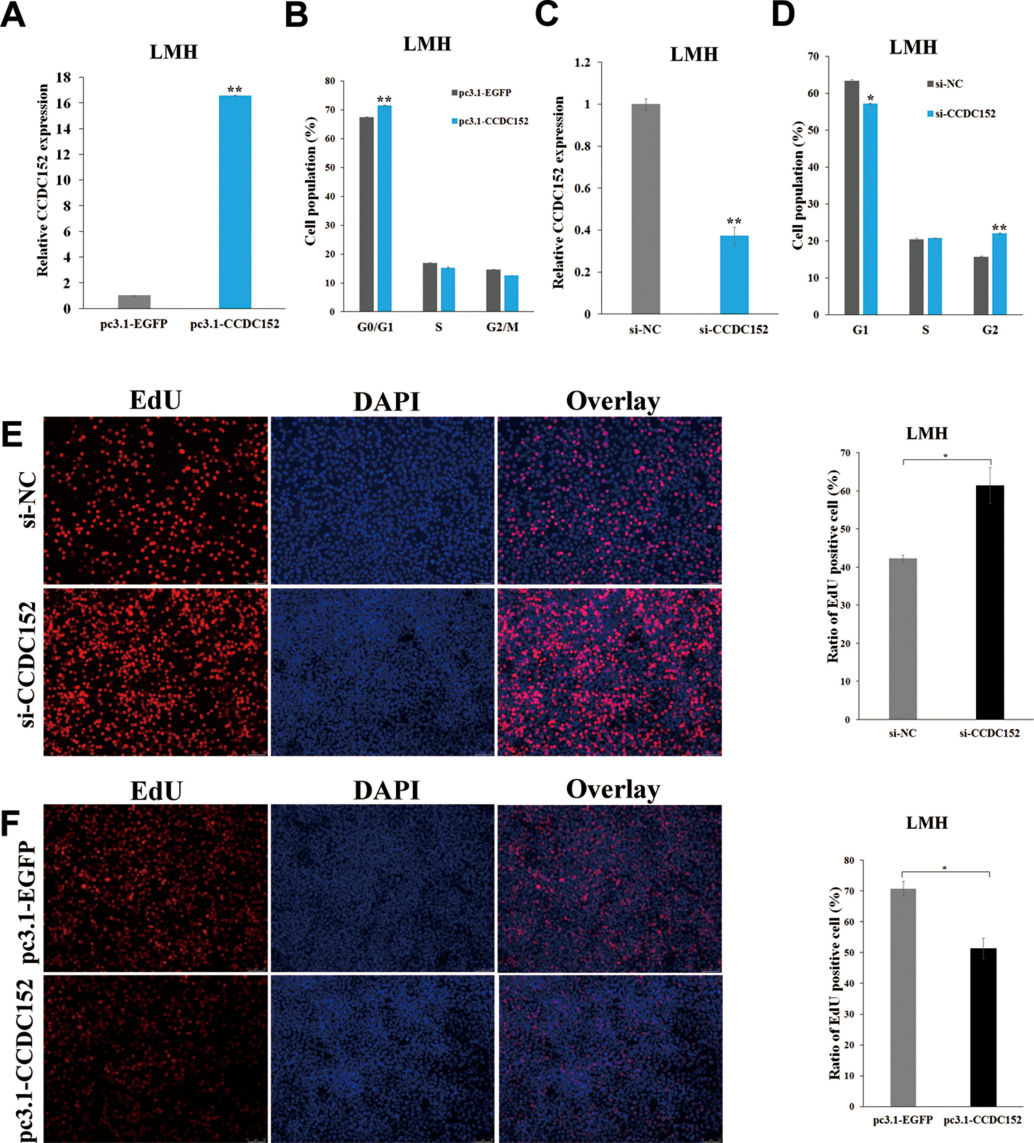
CCDC152 inhibits LMH cell proliferation The relative expression of *CCDC152* after overexpressing *CCDC152* (**A**) and inhibiting *CCDC152* (**C**). Cell cycle analysis of LMH cells 48 h after transfection using flow cytometry (**B, D**). EdU proliferation assays for the cells in 48 h after transfection (Scale bar, 10 μm) and the numbers of proliferative cells were also counted (**E, F**). Compared with the control, per Student's *t-test*, * *P* < 0.05 and ** *P* < 0.01.

We tested the effect of *CCDC152* on cell cycle progression and distribution using flow cytometry. As shown in Figure 7A and 7B, compared with control, overexpression of *CCDC152* in LMH cells promoted a significant arrest in the cell cycle at the G0/G1 phase and reduced the number of proliferating cells. Knockdown of *CCDC152* in LMH cells promoted a significant shift of the cell cycle to the G2 phase from G1 and S phase and increased the number of proliferating cells (Figure 7C, 7D and 7E). Considering these findings with Figure 6G together, we argued that a high level of *CCDC152* expression could inhibit cell proliferation and a low level of *CCDC152* expression would promote cell proliferation through JAK2/STAT signaling pathway.

### Inducing of CCDC152 expression inhibits cell migration

**Figure 8:**
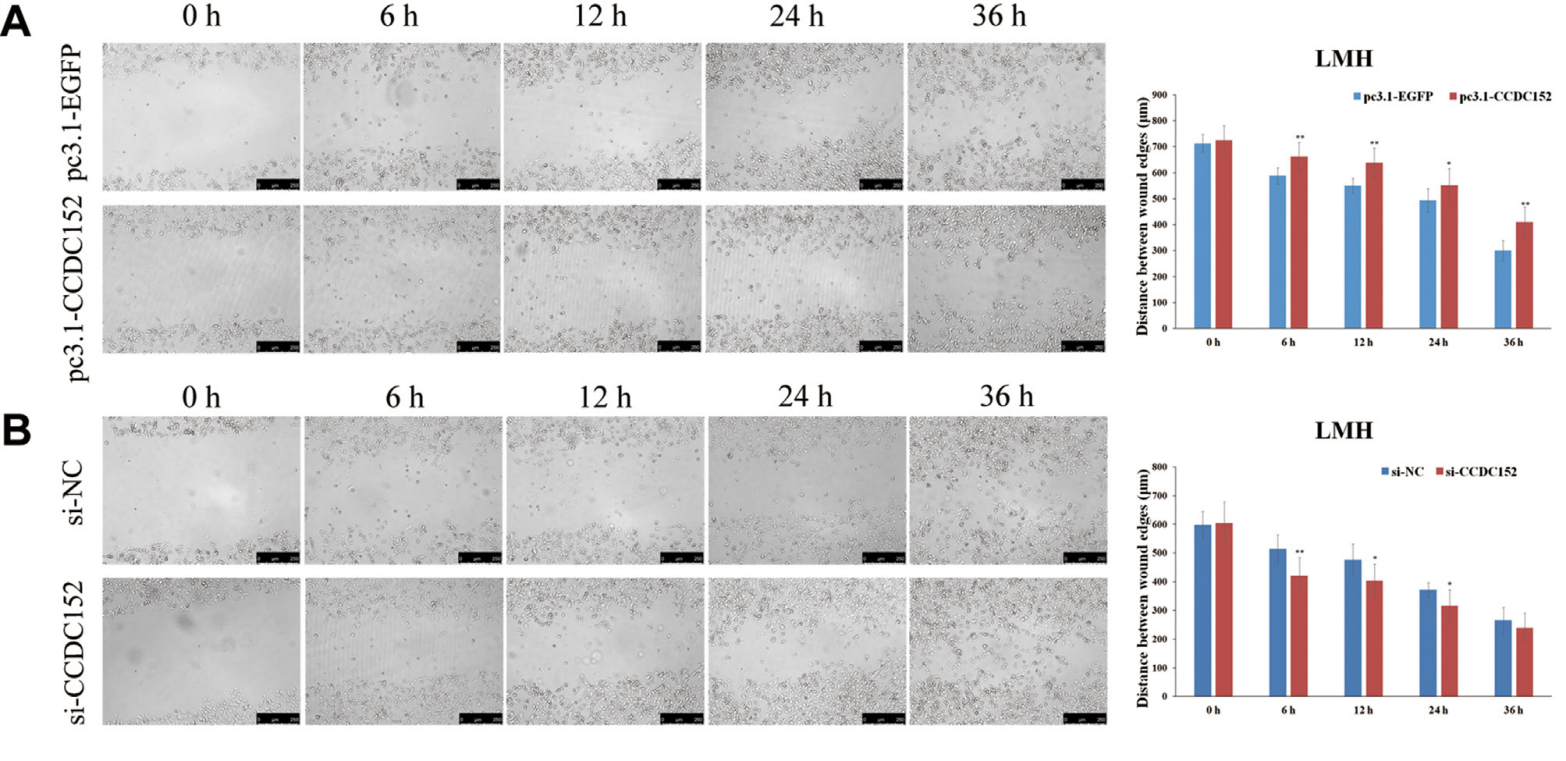
Wound healing assay of LMH cell migration (**A, B**) Representative images of wound healing (cell migration) as described in the Methods, in LMH cells, with induced overexpression *CCDC152* (pc3.1-CCDC152) or knockdown *CCDC152* (si-CCDC152) and control cells transfected with the empty vector (pc3.1-EGFP) or si-NC. Bar graph presentation of the quantitative measurements of cell migration distance in cells overexpressing *CCDC152* (pc3.1-CCDC152) or down-expression *CCDC152* (si-CCDC152) and control cells (pc3.1-EGFP, si-NC). Columns represent the mean of at least four independent experiments and the little vertical bars at the top of the columns represent S.E.M. Compared with the control, per Student's *t*-test, * *P* < 0.05 and ** *P* < 0.01.

**Figure 9:**
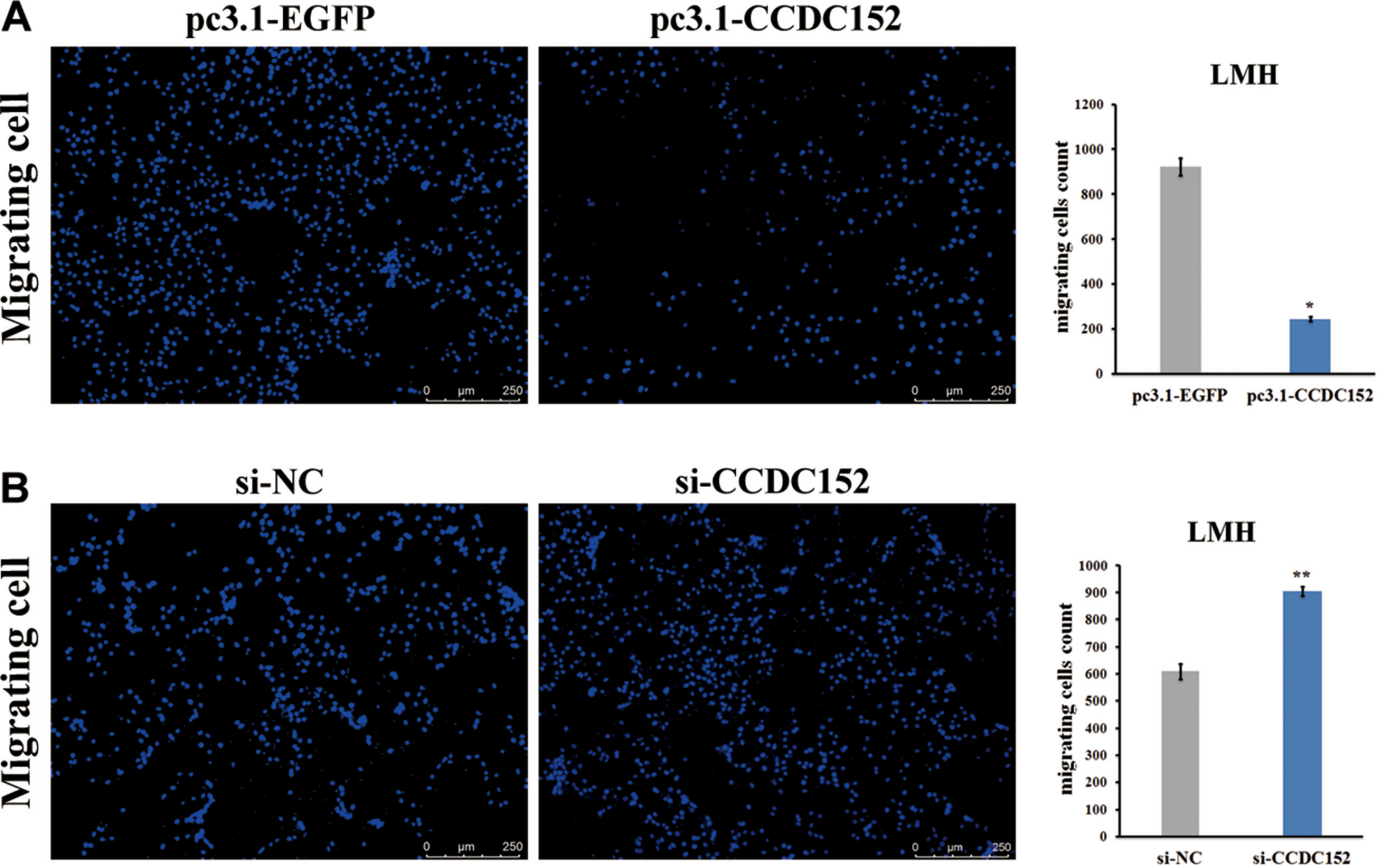
Transwell migration assays of LMH cells (**A, B**) Shown in the left panel are representative microphotograph images of migrating LMH control cells (pc3.1-EGFP, si-NC) or cells transfected with stably introduced expression of *CCDC152* (pc3.1-CCDC152) or interfering oligo of *CCDC152* (si-CCDC152). Scale bar, 50 μm. Columns represent the mean of the counts of migrating cell numbers from at least 3 independent experiments, and the small vertical bars at the top of the columns represent S.E.M. Compared with the control, per Student's *t-*test in both panels A and B, *P < 0.05 and **P < 0.01.

Furthermore, we tested whether *CCDC152* could inhibit the migratory ability of LMH cells by applying the wound healing and the transwell migration assays. Compared with the control cells transfected only with the empty vector, the wound healing ability (migratory ability) of cells was significantly decreased in LMH cells with overexpression of *CCDC152* (Figure 8A). Additionally, compared with control, knockdown of *CCDC152* in LMH cells resulted in a significant increase in the wound healing ability (Figure 8B). Compared to pc3.1-EGFP cells, *CCDC152* cells had a reduced ability to cross the transwell membrane, demonstrated by the weaker staining of the transwells in which they were seeded. Shown in the lower panel is the bar graph presentation of quantitative measurements of cell migration in control LMH cells (pc3.1-EGFP) and LMH cells over-expressing *CCDC152* (pc3.1-CCDC152) (Figure 9A). Compared to si-NC cells, *CCDC152* cells had an increased ability to cross the transwell membrane, demonstrated by the stronger staining of the transwells in which they were seeded. Shown in the lower panel is the bar graph presentation of quantitative measurements of cell migration in control LMH cells (si-NC) and LMH cells knockdown *CCDC152* (si-CCDC152) (Figure 9B).

## DISCUSSION

Bidirectional promoters are a major source of gene activation-associated noncoding RNA [9, 11, 36]. Previous studies have demonstrated that bidirectional promoter is likely to contain CpG islands and many transcriptional factors in the core region, sometimes without the TATA box [37, 38]. The present study clearly illustrates that *GHR-AS-I6* and *CCDC152* constitute a classical head-to-head pair that shares a prototypical bidirectional promoter for the first time. In addition, the intergenic distance between *GHR-AS-I6* and *CCDC152* is 504 bp or even less (102 bp) in some circumstances. Also, this region contains a TATA box and harbors a CCAAT box for NFYB, which is frequently found in bidirectional promoters. Moreover, this region does not contain a CpG island (data not shown), which is sometimes presented in bidirectional promoters. The nuclear sequence-specific transcription factor NF-Y complex is a trimer that binds to the CCAAT box [35, 39] and participates in regulating proliferation by controlling the expression of genes required for cell cycle progression [33, 40] and transcriptional initiation of various genes [13, 34, 41, 42]. In the present study, we demonstrate that the classic *GHR-AS-I6-CCDC152* pair shares a prototypical bidirectional promoter that is regulated by NFYB/NFYC complex. Considering that only a few examples of such bidirectional promoter have been identified and mechanistically investigated to date, this study also represents a useful paradigm for analyzing the transcriptional regulation of bidirectional promoter-driven gene pairs.

It has been reported that *CCDC* genes play important roles in the process of gene transcription [14, 29, 43]. In this study, the chicken *CCDC152* can regulate the *GHR-AS-I6-CCDC152* bidirectional promoter through JAK/STAT3/5 signaling pathway, which have been reported by other researchers to mediate genes′ transcription [44, 45] and participate in cells proliferation, growth, differentiation, migration and invasion [46, 47]. Previous studies have shown that *CCDC* genes are relative to cellular progression, such as the cell cycle, proliferation and migration [28, 29, 48]. Notably, one of these genes, *CCDD152*, was previously indicated to express in chicken liver and leg muscle tissues (Supplementary Figure 2). So we proposed that the chicken *CCDC152* may play a pivotal role in cell proliferation and migration mediate JAK2/STAT signaling pathway. In this study, we investigated the suppressor function of *CCDC152* in LMH cells. As expected, the overexpression of *CCDC152* suppressed LMH cell development, including cell proliferation and migration by down-regulating *JAK2*, *STAT3* and *STAT5* expression. All these results show that CCDC152 plays pivotal role in inhibiting cell proliferation and migration by influencing JAK2/STAT signaling. However, further studies are needed to confirm whether the CCDC152 protein directly or indirectly activate/inactivate JAK2/STAT signaling to mediate cell proliferation, e.g. how the effect of CCDC152 overexpression blocking the STAT3/STAT5B pathway occurs on cells proliferation and whether CCDC152 regulates JAK2/STAT phosphorylation and their protein expressions.

**Figure 10:**
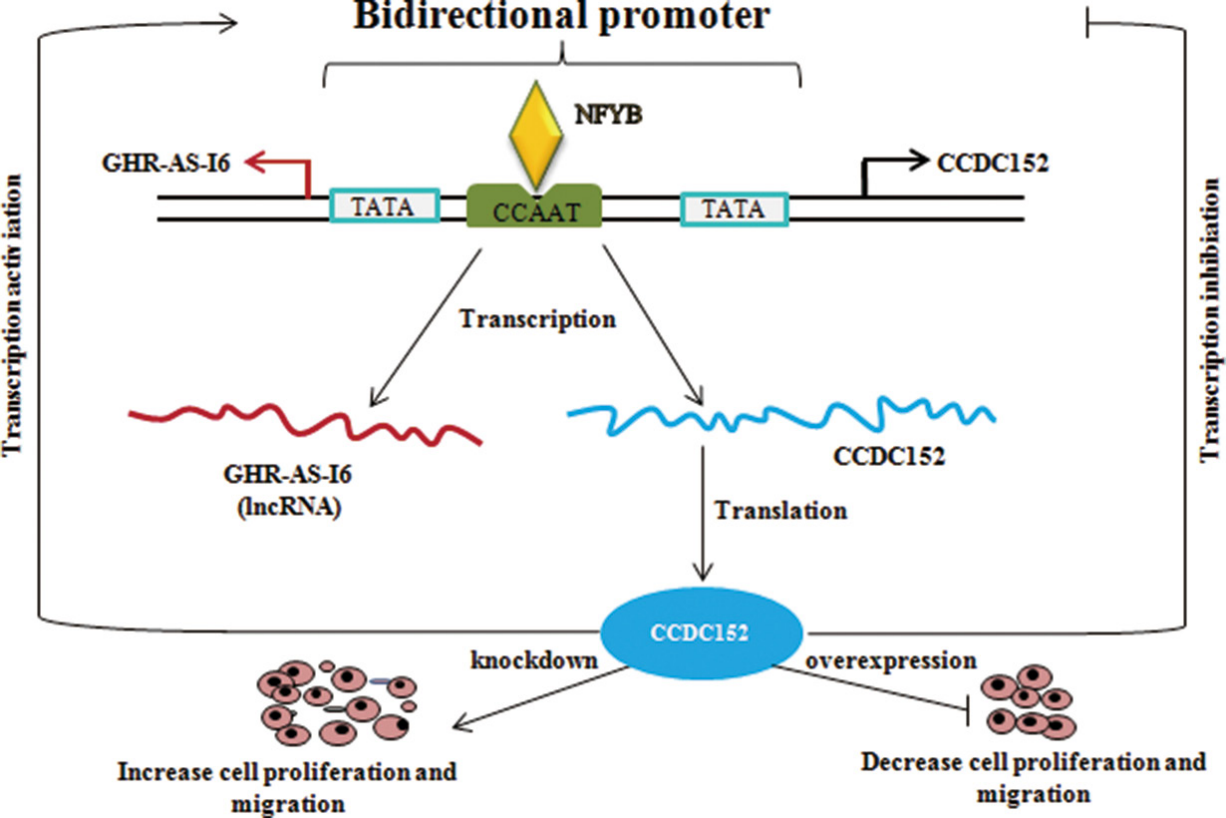
The NFYB controls chicken GHR-AS-I6-CCDC152 bidirectional promoter in LMH cells The low level of *CCDC152* can positively regulate the bidirectional promoter to transcribe *GHR-AS-I6* and *CCDC152* (left), and in contrast, the high level of *CCDC152* will inhibit the activity of this bidirectional promoter (right). Therefore, *CCDC152* can balance the expression level of itself to regulate cell proliferation and migration.

In summary, as shown in Figure 10, NFYB combined with NFYC could target the CCAAT box of the bidirectional promoter to trigger both *GHR-AS-I6* and *CCDC152* transcriptions. A high level of CCDC152 protein can not only suppress the bidirectional promoter activity, but also inhibit cell proliferation and migration; conversely, a low level of CCDC152 protein can increase bidirectional promoter activity, as well as promote cellular proliferation and migration by mediating JAK2/STAT3/5 signaling pathway.

## MATERIALS AND METHODS

### Ethics statement

The Animal Care Committee of South China Agricultural University (Guangzhou, People's Republic of China) approved this study. The animals involved in this study were humanely sacrificed as necessary to ameliorate suffering.

### Computational analyses

The chicken *GHR* and *CCDC152* genes sequences were downloaded from NCBI database. The potential promoter region and transcriptional start sites of *GHR-AS-I6-CCDC152* were predicted by online softwares PromoterScan (http://www-bimas.cit.nih.gov/molbio/proscan/) and Fruitfly (http://linux1.softberry.com/berry.phtml?topic=fgenesh&group=programs&subgroup=gfind). The transcriptional start site of *CCDC152* was decided by *CCDC152* mRNA (NCBI: XM_015277374.1). The online tools Jaspar (http://jaspar.genereg.net/) and PROMO (http://alggen.lsi.upc.es/cgi-bin/promo_v3/promo/promoinit.cgi?dirDB=TF_8.3) were used to predict the TATA box and transcriptional factor binding sites.

### Experimental animals

White Recessive Rock (WRR) chicken at seven-week-old was obtained from Experimental Poultry Farm of South China Agricultural University (Guangdong, China). Liver tissue samples were taken from the WRR and stored in liquid nitrogen before use.

### Extraction of genomic DNA

Genomic DNA was extracted from the WRR chicken liver tissue samples following standard saturated phenol-chloroform extraction procedures. After extraction, DNA quantity was assessed using NanoDrop 2000 spectrophotometer (Thermo Scientific).

### Rapid amplification of cDNA 5′ ends (5′-RACE)

5′-RACE was conducted using the PrimeScript™ II 1st Strand cDNA Synthesis Kit (Takara, Japan) and two steps of nested PCR. Primers were designed using Oligo7 so that a relatively large section of the non-coding region was amplified in addition to the 5′ untranslated region. Briefly, total RNAs were extracted from WRR chicken liver and leg muscle tissues using the Trizol reagent (Invitrogen, USA), and were reversely transcribed to obtain first-strand cDNA with the universal primer AUP1 onto the 5′ end following the PrimeScript™ II manual (Takara, Japan). There were a total of 6 tubes, each containing 20 μL cDNA treated with RNase H. A total of 75 μL of the reaction mixture was added to each tube, comprising 20 μL reverse transcription product, 15 μL 5 × Hybrid RNA Degeneration Buffer, 1 μL RNase H (60 U ∙ μL-1), and 39 μL DEPC water. Then, the solution was incubated at 30°C for 1 h. Thereafter, the PCR product purification kit (Magen, China) was used to purify the RNase H treatment product. When the product was purified, the 2 tubes were combined into 1 tube through the cDNA of the enzyme solution, and 20 μL elution buffer per tube was added. The purified cDNA 10 μL, 5 x TdT Buffer 10 μL, 0.1% BSA 5 μL, 100 mmol ∙ L-1 dCTP 0.5 μL, TdT 1.5 L (ThermoFisher, USA), and double distilled water were added up to 50 μL, and the solution was incubated at 37 °C for 5 h for TdT polyadenylation (ThermoFisher, USA). For each replicate, 2 tubes were used. After the end of treatment, the product was purified with PCR product purification kit (Magen, China), and the 2 tubes of products were combined into 1 tube for purification, washed, then eluted in with 30 μL of elution buffer, and stored at –30°C.

The first round of PCR amplification was then examined using upstream universal outside primer AP1 and *CCDC152* or *GHR-AS-I6* gene-specific outside primer. The nested PCR based amplification was then performed using upstream universal inside primer AP2 and *CCDC152* or *GHR-AS-I6* gene-specific inside primers (Supplementary Table 1). The PCR procedure was as follows: 95°C for 3 min for degeneration; 95°C for 40 s, 72°C for 3 min for two cycles; 95°C for 40 s, 70°C for 3 min for two cycles; 95°C for 40 s, 68 °C for 3 min for two cycles; and 95°C for 40 s, 66°C for 50 s, 72°C for 3 min, for 30 cycles. PCR products were gel-purified using a 1.5% gel and PCR product purification kit (Magen, China), cloned into pMD18T Vector (Takara, Japan), and sequenced by Shanghai Sangon Biotech (Guangzhou, China). The RACE sequences were subsequently compared with the genomic sequences of *CCDC152* and *GHR-AS-I6* to confirm gene identity, exon usage, and location of the transcription start sites.

### Cell culture

The chicken LMH cells line was donated by Harbin Veterinary Research Institute Chinese Academy of Agricultural Science (Heilongjiang, China). We cultured them in high-glucose Dulbecco′s modified Eagle′s medium (Gibco, Grand Island, NY, USA) with 10% heat-inactivated fetal bovine serum (Invitrogen, USA), penicillin (100 IU ∙ mL-1), and streptomycin (100 μg ∙ mL-1) (Invitrogen, Carlsbad, CA, USA). All cells were maintained at 37°C in 5% CO2.

### Luciferase reporter constructs and reporter assays

The qualitative analyses of the bidirectional promoter GFP luciferase reporter pEGFP-CCDC152-P1180 and pEGFP-GHR-AS-I6-P1180 were generated by cloning the corresponding intergenic region between *GHR* 3′ flanking region and *CCDC152* into the pEGFP-N1 vector to substitute its CMV region, and pLinker-EGFP was the result of deletion of the CMV region of pEGFP-N1 using Ω-PCR as reported elsewhere [49]. The primer sequences and the restriction enzymes used are listed in Supplementary Table 1. LMH cells were inoculated into 24 wells, approximately 2.5 × 10^5^ cells per well. According to the manufacturer's instructions for Lipofectamine3000, pEGFP-CCDC152-P1180 and pEGFP-GHR-AS-I6-P1180 were used for transfection of LMH cells, and a positive (pEGFP-N1 transfected cells) and negative control group (pLinker-EGFP transfected cells) were created. After 48 h of transfection, fluorescence was observed under Nikon Eclipse microscopy (Nikon, Tokyo, Japan).

The luciferase reporters CCDC152-P1180, CCDC152-P874, CCDC152-P504, CCDC152-P317, CCDC152-P102, GHR-AS-I6-P1180, GHR-AS-I6-P874, GHR-AS-I6-P504, GHR-AS-I6-P317 and GHR-AS-I6-102 were generated by cloning the corresponding intergenic region between *GHR* 3’ flanking region and *CCDC152* into the pGL3-basic vector using PCR or deletion Ω-PCR [49]. The primer sequences and the restriction enzymes used are also listed in Supplementary Table 1. For luciferase reporter assays, cells were seeded in triplicate into 24-well plates and co-transfected with the indicated reporter plasmid, pRL-TK vector (Promega) encoding Renilla luciferase and the pcDNA3.1 empty vector or a transcription factor expression vector using Lipofectamine 3000 (Invitrogen, USA). Then 48 h after transfection, cells were lysed with passive lysis buffer, and luciferase activity was measured using the Dual-Luciferase Assay System (Promega) according to the manufacturer's protocols.

### Site mutation constructs and transient transfection assays

Various progressive deletion mutants (M1-M8, M1′-M8′) were further constructed using either a DNA blunting kit (Takara, Japan) with restriction enzyme digestion and ligation, or the Site-Directed Mutagenesis Kit (TOYOBO, Japan) with reverse PCR amplification and ligation, according to the manufacturers’ instructions. Constructs with point mutation(s) at putative transcription factor binding sites were generated using Ω-PCR [49]. The primer sequences and the restriction enzymes used are listed in Supplementary Table 1. All constructs were validated by direct sequencing, and the transient transfection assays were conducted as above.

### Overexpression and siRNA transfection

The overexpression constructs pc3.1-CCDC152 and pc3.1-NFYB were the results of their CDS-sequences inserted into a pcDNA-3.1 vector. All siRNAs were chemically synthesized by Shanghai GenePharma (Shanghai, China). The sequences of the siRNAs used are listed in Table 1. The perfect concentration of pc3.1-CCDC152, pc3.1-NFYB (Supplementary Figure 3) and siRNAs (Supplementary Figure 4) were transfected into the indicated cells using Lipofectamine 3000 (Invitrogen, USA) according to the manufacturer's instructions. Cells were then collected and subjected to analysis 48 h after transfection. The experiments were repeated at least in triplicate.

**Table 1:** siRNAs targeting chicken *CCDC152* and *NFYB*

Name	Sequence (5′–3′)
si-NC	UUCUCCGAACGUGUCACGUTT
	ACGUGACACGUUCGGAGAATT
si-CCDC152	GAAACACCAUAAGAGACUUTT
	AAGUCUCUUAUGGUGUUUCTT
si-NFYB	CCGAUUGCAAACGUGGCAATT
	UUGCCSCGUUUGCAAUCGGTT

### RNA isolation and qRT-PCR

Total RNA isolation was conducted with RNA Isolation Tissue Kit (BIOMEGA), and reverse transcription was performed with PrimerScript^TM^ II Kit (TaKaRa, Japan) to prepare cDNA. The resulting cDNA was used in qRT-PCR reactions with *iTaq*™ *universal* *SYBR*® Green (Bio-Rad) on a MasterCycle Realplex2 SYSTEM (Eppendorf) and analyzed as described. The sequences of the primers used are provided in Supplementary Table 1.

### Chromatin immunoprecipitation assay

Chromatin immunoprecipitation (ChIP) assays were performed according to the ChIP Assay Kit (Beyotime, Shanghai, China). Briefly, LMH cells were treated with 37% formaldehyde and incubated for 10 min to generate DNA-protein cross-links. Cell lysates were then sonicated to generate chromatin fragments of 200–500 bp and immunoprecipitated with antibodies against anti-NFYB and anti-NFYC (LSBio, USA) or IgG antibody (Beyotime, Shanghai, China) as negative control (NC). The antibody-bound complex was precipitated by Protein A+G Agarose beads. The DNA fragments in the immunoprecipitated complex were released by reversing the cross-linking at 65°C for 4 hour and purified DNA was analyzed by PCR and qRT-PCR with *iTaq*™ *universal* *SYBR*® Green (Bio-Rad). The ΔCt was calculated as ΔCt (normalized ChIP) = [Ct (ChIP) – Ct (Input)]. The % input was shown as 2 ^[-ΔCt(normalized ChIP)]^. The primers specific for the P102 region of the bidirectional promoter containing CCAAT box were shown in Supplementary Table 1.

### Western blot analysis

For Western blotting, 30 mg of fresh frozen treated cells were lysed in ROPA lysis buffer (Cwbiotech, China) containing 1% Protease Inhibitor Cocktail (Cwbiotech, China). An equal amount of protein, approximately 30 μg, was separated by sodium dodecyl sulfate polyacrylamide gel electrophoresis (SDS-PAGE) and transferred onto the poly vinylidene flouride (PVDF) membrane. After blocking with 5% skimmed milk, the PVDF membrane was incubated with anti-CCDC152 (Santa, USA), anti-NFYB and anti-NFYC (LSBio, USA) antibodies, respectively. After washing three times with Tris-Buffered Saline and Tween 20, the membrane was incubated with horseradish peroxidase-linked secondary anti-goat or anti-rabbit IgG antibody (Santa, USA) at room temperature for 1 h, followed by visualization using an ECL detection kit (Millipore, Billerica, MA). Anti-β-actin antibody was purchased from Abcam and used as loading controls. An ImageMaster DVS system was used to calculate the relative mean gray values (A) of the target product and β-actin. Expression index (I) of the target produce was calculated using the formula of I = A produce/A β-actin.

### Cell proliferation assay

Cell cycle analysis: Cells transfected with pc3.1-CCDC152 and si-CCDC152, and control cells were harvested 72 h after culture, washed with cold phosphate buffered saline (PBS), and fixed in 1 mL of cold 70% ethanol. After overnight incubation at –20°C in ethanol, cells were washed in PBS and suspended in 500 mL propidine iodide (PI) for 30 min before flow cytometry. Populations in G1, S and G2 phases were measured by flow cytometry (Beckman, CA), and the data were analyzed using the Multicycle-DNA Cell Cycle Analysis Software. The measurements were performed four times.

DNA synthesis in proliferating cells was determined by EdU assay, using Cell-Light^TM^ EdU Appolo567 In Vitro Kit (Ribobio, China) following manufacturer's instructions. Treated cells were observed using fluorescence microscope (Leica, Germany). Experiments were repeated at least three times in duplicates.

### Wound-healing assay and transwell migration assay

A wound healing assay was carried out to detect the migration of cells, and the three kinds of transfectant cells were cultured in 24-well plates until confluent. The cell layer was wounded using a sterile tip. After incubation for 0 to 48 h, cells were photographed under an inverted microscope (Leica, Germany). Experiments were carried out four times. The distance between the two edges of the scratch (wound width) was measured at 8 sites (100 × magnification) in each image. The cell migration distance was measured using ImageJ by measuring the wound width at 0 h and at each time point, and then dividing by two.

For migration assay, 200 μL of the pc3.1-CCDC152, si-CCDC152 and NC cells (transfected for 12 h) in serum-free DMEM were seeded on upper migration chambers (24-well plats, 8-μm pore size. Corning Incorporated Costar, USA) at a density of 2 × 10^5^ cells/well, and incubated at 37 °C and 5% CO_2_. A volume of 700 μL of DMEM containing 10% fetal bovine serum was added in the lower chamber. After incubation for 24 h, the migrating cells in the upper migration chambers were fixed with 800 μL 4% paraformaldehyde at room temperature for 30 min, and then stained with 800 μL 0.1% DAPI (Ribobio, China) at room temperature for 30 min. The unpenetrated cells on the upper chamber were gently scraped from the surface with cotton swabs. Six visual fields (100 × magnification) were selected for each chamber and photographed under an inverted microscope (Leica, Germany) and the number of penetrated cells in these six fields was counted by ImageJ software. The mean value of each group was recorded.

### Statistical analyses

The relative expression level of the target gene was calculated with the 2^-ΔΔCt^ method and error bars represent the SEM from at least three repeats. To know the significance of the observed differences, *P-values* were assessed using two-tailed unpaired, Student's *t-test* with *P-values* < 0.05 or less considered to be statistically significant.

## SUPPLEMENTARY MATERIALS FIGURES AND TABLES





## Abbreviations

CCDC152: coiled-coil domain-containing protein 152
GHR: growth hormone receptor
GHR-AS-I6: GHR antisense transcript intron 6
LMH: chicken hepatocellular carcinoma cell line
qRT-PCR: quantitative real-time polymerase chain reaction
ChIP: chromatin immunoprecipitation
NFYB/NFYC: nuclear transcription factor Y subunit beta/gamma
JAK: Janus kinase
STAT: signal transducer and activator of transcription
