# Supplementary material for: Chicken CCDC152 shares an NFYB-regulated bidirectional promoter with a growth hormone receptor antisense transcript and inhibits cells proliferation and migration
Source: Oncotarget. 2017 Sep 20;8(48):84039–53. doi: 10.18632/oncotarget.21091 (PMC5663575; doi:10.18632/oncotarget.21091)
Supplement: Supplementary file 1 [file oncotarget-08-84039-s001.pdf]

Chicken *CCDC152* shares an NFYB-regulated bidirectional promoter with a *growth hormone receptor* antisense transcript and inhibits cells proliferation and migration

SUPPLEMENTARY MATERIALS

Supplementary Table 1: Primers used in this study. See Supplementary_Table_1

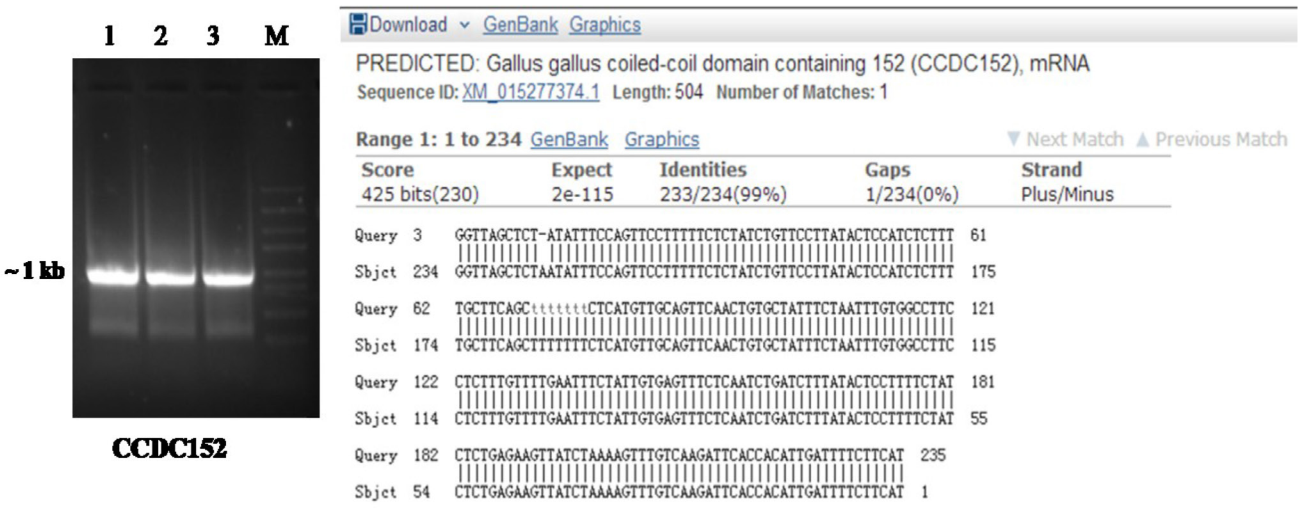

Supplementary Figure 1: Different 5′-RACE PCR products of CCDC152 were amplified from WRR chicken liver and leg muscle tissues. In the left side, the Trans5K DNA Marker consists of 300 bp, 500 bp, 800 bp, 1000 bp, 1500 bp, 2000 bp, 3000 bp, 5000 bp. The 1 and 2 lanes were from liver tissues, and the 3 lane comes from leg muscle tissue. The right side shows the alignment between the sequencing result and the NCBI Database by NCBI BLAST.

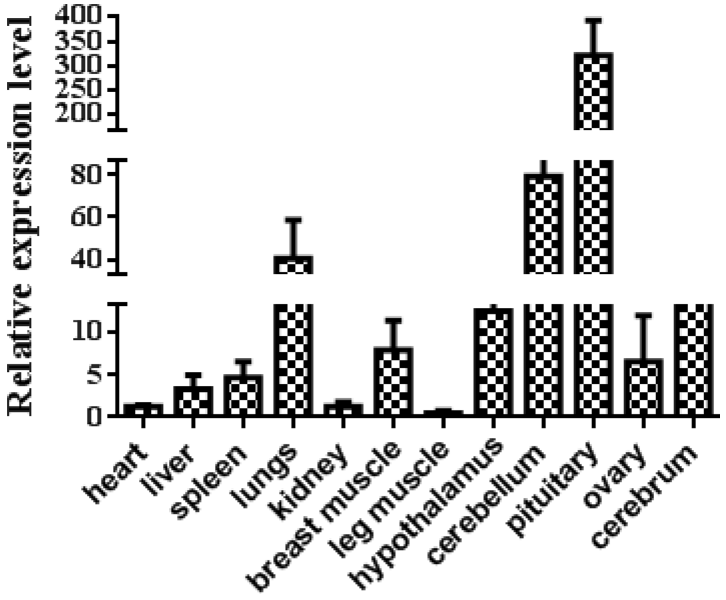

Supplementary Figure 2: The tissue expression profile of chicken CCDC152. The chicken CCDC152 is highly expressed in pituitary, cerebellum, lungs and cerebrum tissues, and low expression level was seen in liver, heart and leg muscle tissues.

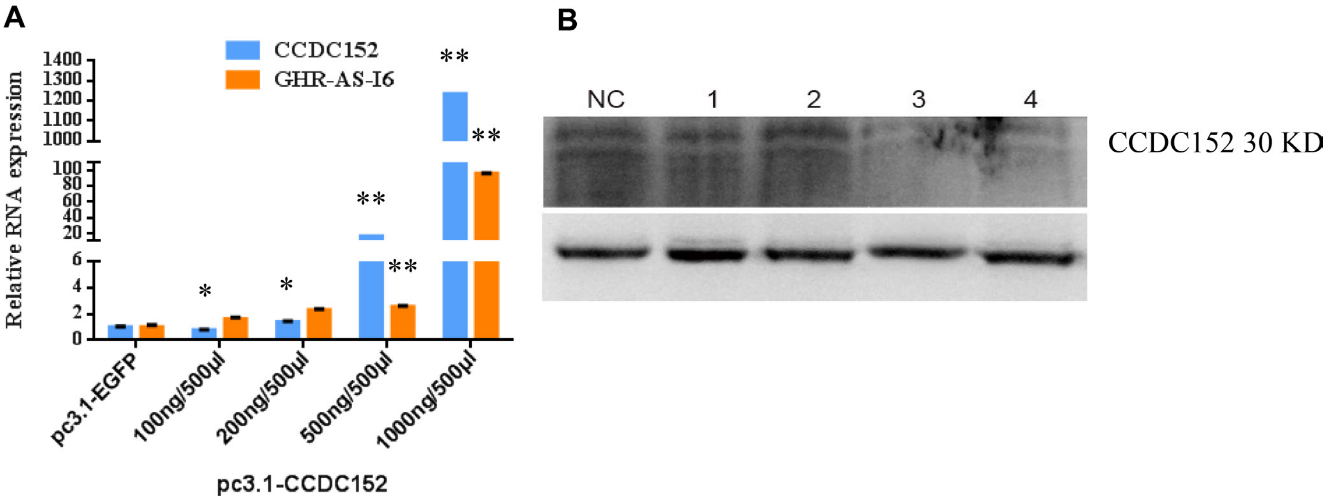

Supplementary Figure 3: qRT-PCR and western blot analysis for determination of *CCDC152*-best concentration after *CCDC152* overexpression. (**A**) Relative expression levels of *CCDC152* and *GHR-AS-I6* were shown after transient transfection with different pc3.1-*CCDC152* concentrations (100 ng/500 µl, 200 ng/500 µl, 500 ng/500 µl and 1000 ng/500 µl). (**B**) Western blot assay was used to detect the expression level of CCDC152 protein after CCDC152 overexpression. For NC, LMH cells were transfected with pc3.1-EGFP, and 1-4 lanes represented LMH cells treated with different concentrations of pc3.1-CCDC152 (100 ng/500 µl, 200 ng/500 µl, 500 ng/500 µl and 1000 ng/500 µl). We selected the 1000 ng/500 µl of pc3.1-CCDC152 as best concentration for treating cells. Student's t test was performed, *$P < 0.05$ and **$P < 0.01$ Vs. NC.

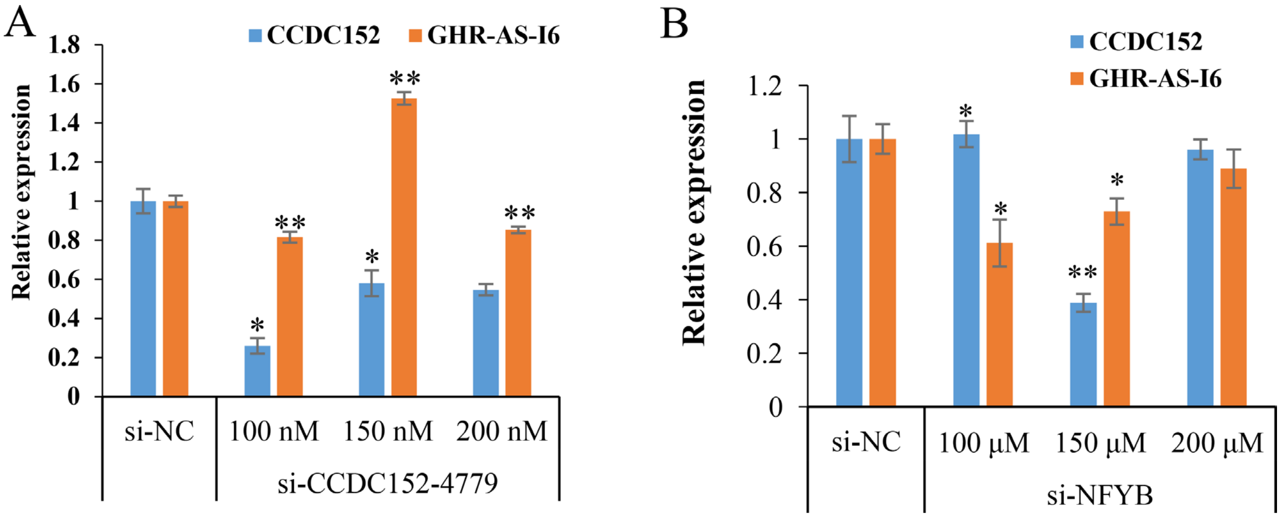

Supplementary Figure 4: Detectable the best concentration of inhibiting CCDC152 and NFYB. (**A**) Relative expression levels of CCDC152 and GHR-AS-I6 were shown after transient transfecting with different concentrations of si-CCDC152 (100 nM, 150 nM and 200 nM). According to the result, we have chosen 100 nM concentration of si-CCDC152 for transfecting cells. (**B**) Relative expression levels of CCDC152 and GHR-AS-I6 were shown after transient transfection with different concentrations of si-NFYB (100 nM, 150 nM and 200 nM). Therefore, we have chosen 150 nM concentration of si-NFYB to achieving best results. Student's t test was performed, *$P < 0.05$ and **$P < 0.01$ Vs. NC.
