# Supplementary material for: Chicken CCDC152 shares an NFYB-regulated bidirectional promoter with a growth hormone receptor antisense transcript and inhibits cells proliferation and migration
Source: Oncotarget. 2017 Sep 20;8(48):84039–53. doi: 10.18632/oncotarget.21091 (PMC5663575; doi:10.18632/oncotarget.21091)
Supplement: Supplementary file 2 [file oncotarget-08-84039-s002.docx]

**Supplementary Table 1: Primers used in this study**

| **ID** | **Primer name** | **Primer sequence (5'to3')** | **Application** |
| --- | --- | --- | --- |
| universal primer | AUP1 | GGCCACGCGTCGACTAGTACTTTTTTTTTTTTTTTTTT | first-strand cDNA |
| upstream universal outside primer | AP1 | AAGCAGTGGTATCAACGCAGAGTACGCGGGGGG | 5′-RACE |
| upstream universal inside primer | AP2 | AAGCAGTGGTATCAACGCAGAGT |  |
| 5′-CCDC152 | GSP | GTGCTGTAGTTTCCTTCGATAGATACT |  |
|  | Nested-GSP | TTCTGTTTCTCTTCATCTTGAGTTTTC |  |
| 5′-GHR-AS-I6 | GSP | TGAAGTAGTGAGGTTTCCATCAGTCCA |  |
|  | Nested-GSP | GTGCCAAAGTAAACAGCAGATGCCGAA |  |
| pLinker-EGFP | pEGFP-Linker-F | TACCGCCATGCATTAGTTATTAGAGCTGGTTTAGTGAAC | GFP signal detection |
|  | pEGFP-Linker-R | GTTCACTAAACCAGCTCTAATAACTAATGCATGGCGGTA |  |
| pEGFP-CCDC152-P1180 | pEGFP-Ase I-F | CGC*ATTAAT*GCTCCCTCATTTCGTTTTATT |  |
|  | pEGFP-Xho I-R | TA*CTCGAG*GCACTGCTGCACATAAACCTCACCG |  |
| pEGFP-GHR-AS-I6-P1180 | pEGFP-Ase I-F | GCC*ATTAAT*CAGCTTTGAAACAGCATG |  |
|  | pEGFP-Xho I-R | CGC*CTCGAG*GCTCCCTCATTTCGTTTTTATT |  |
| pGL3-CCDC152 | P1180-KpnI-F | GAT*GGTACC*GCTCCCTCATTTCGTTTTATT | deletion constructs |
|  | P1180-XhoI-R | TA*CTCGAG*CAGCTTTGAAACAGCATG |  |
|  | P874-Xho I-R | GA*CTCGAG*CCCAAATGTAAGCAGAGCACA |  |
|  | P504-Xho I-R | ATA*CTCGAG*GCAACAGTTGAATCTCCGTTAGAT |  |
|  | P317-Xho I-R | ATA*CTCGAG*GCACTGCTGCACATAAACCTCACCG |  |
|  | P102-Kpn I-F | GAT*GGTACC*GCTCCCTCATTTCGTTTTTATTTAC |  |
|  | P102-Xho I-R | CGC*CTCGAG*TTCCAATACAGATAAAGCAT |  |
| pGL3-GHR-AS-I6 | P1180-KpnI-F | ATA*GGTACC*CAGCTTTGAAACAGCATG |  |
|  | P874-Kpn I-F | ATA*GGTACC*CCCAAATGTAAGCAGAGCACA |  |
|  | P504-Kpn I-F | ATA*GGTACC*GCAACAGTTGAATCTCCGTTAGAT |  |
|  | P317-Kpn I-F | ATA*GGTACC*GCACTGCTGCACATAAACCTCACCG |  |
|  | P317-Xho I-R | GAT*CTCGAG*GCTCCCTCATTTCGTTTTTATT |  |
|  | P102-Kpn I-F | GA*GGTACC*TTCCAATACAGATAAAGCAT |  |
|  | P102-Xho I-R | CGCCTCGAGGCTCCCTCATTTCGTTTTTA |  |
| Promoter site mutation | M1F/M1'F | GTAAAAACAGAAATTGTGTTGCTGGCGCGAAGCTATATGGGTGTGCTTCA | mutation constructs |
|  | M1R/M1'R | TGAAGCACACCCATATAGCTTCGCGCCAGCAACACAATTTCTGTTTTTAC |  |
|  | M2F/M2'F | CAAATGTGTTTTAGTAACATACCAGCGACGAAGTAGCTCGTATGTTTTAA |  |
|  | M2R/M2'R | TTAAAACATACGAGCTACTTCGTCGCTGGTATGTTACTAAAACACATTTG |  |
|  | M3F/M3'F | GAAAATTAATAGGTCTATTTTTTAGCGACCAGGTGGTCACTGGATGAAAT |  |
|  | M3R/M3'R | ATTTCATCCAGTGACCACCTGGTCGCTAAAAAATAGACCTATTAATTTTC |  |
|  | M4F/M4'F | CCACTTGAAAAGCTTACCACGAGCCATCTGAATATCTATGGATAGCAAT |  |
|  | M4R/M4'R | ATTGCTATCCATAGATATTCAGATGGCTCGTGGTAAGCTTTTCAAGTGG |  |
|  | M5F/M5'F | TAAAGCTACATACAAATTTTCGGCACTGAAATCCACATTAATATCAGTGA |  |
|  | M5R/M5'R | TCACTGATATTAATGTGGATTTCAGTGCCGAAAATTTGTATGTAGCTTTA |  |
|  | M6F/M6'F | GAAATTGCAAGATGCATCTGACGGGTGACATATGAGTGTTTCTAAAAAT |  |
|  | M6R/M6'R | ATTTTTAGAAACACTCATATGTCACCCGTCAGATGCATCTTGCAATTTC |  |
|  | M7F/M7'F | AGGCTTCATCAAACAATAATGAGCCGTGAGATAAAGCATATATTCTGGAC |  |
|  | M7R/M7'R | GTCCAGAATATATGCTTTATCTCACGGCTCATTATTGTTTGATGAAGCCT |  |
|  | M8F/M8'F | ATCATCAGTTCTGCATAGGAATTCGGACGGTCTGTTACATATAGTAAATA |  |
|  | M8R/M8'R | TATTTACTATATGTAACAGACCGTCCGAATTCCTATGCAGAACTGATGAT |  |
|  | CCDC152-P102-Kpn I-F | GAT*GGTACC*GCTCCCTCATTTCGTTTTTATTTAC |  |
|  | CCDC152-P102-mut-Xho I-R | GCG*CTCGAG*TTGATCAACAGATAAAGCAT |  |
|  | GHR-AS-I6-P102-mut-Kpn I-F | GAT*GGTACC*TTGATCAACAGATAAAGCAT |  |
|  | P102-Xho I-R | CGCCTCGAGGCTCCCTCATTTCGTTTTTA |  |
| CCDC152 | CCDC152-EcoR I-F | CGC*GAATTC*ATGAAGAAAATCAATGTGGT | overexpression constructs |
|  | CCDC152-Xho I-R | TTA*CTCGAG*ACCGCCTCCGTTTCAAGT |  |
| NFYB | NFYB-EcoR I-F | GCC*GAATTC*ATGCATTAATCTAATGAGGTCA |  |
|  | NFYB-Xho I-R | CGC*CTCGAG*CTATTTGTAGTCTGTGAAATC |  |
| GAPDH | GAPDH-F | ATGGCATCCAAGGAGTGA | ChIP-PCR and -qRT-PCR |
|  | GAPDH-R | GGGAGACAGAAGGGAACAG |  |
| P102 | P102-F | GCTCCCTCATTTCGTTTTTATTTAC |  |
|  | P102-R | TTCCAATACAGATAAAGCAT |  |
| CCDC152 | CCDC152-qPCR-F | CAAAACAAAGAGGAAGGCCACAA | qRT-PCR |
|  | CCDC152-qPCR-R | ACCGCCTCCGTTTCAAGTGC |  |
| GHR-AS-I6 | GHR-AS-I6-qPCR-F | TTGCTAATGTTTCTGTTCTGTG |  |
|  | GHR-AS-I6-qPCR-R | GGGTCAATCCCTTTAATCTTT |  |
| NFYA | NFYA-qPCR-F | GGAGCCAACACCAATACCAC |  |
|  | NFYA-qPCR-R | TCCAGGCAAAGGTATTCGC |  |
| NFYB | NFYB-qPCR-F | TGAGGTCAAAAGTGCCAAGAG |  |
|  | NFYB-qPCR-R | CCAATGTAATCTCCAGCAATTCC |  |
| NFYC | NFYC-qPCR-F | AGTTCAGCCAGTTCACAGAC |  |
|  | NFYC-qPCR-R | CTGCTCAATCCCCTGTCAC |  |
| JAK2 | JAK2-qPCR-F | TTTCAGGCCGTCATTTAGGG |  |
|  | JAK2-qPCR-R | TTGGGTCCCGATCTTCAAAG |  |
| STAT1 | STAT1-qPCR-F | GATATTCCCAAAGACAACGCC |  |
|  | STAT1-qPCR-R | GATGGGTGGACTTCAGATACAG |  |
| STAT2 | STAT2-qPCR-F | ACATTCACATCGACAGGGAC |  |
|  | STAT2-qPCR-R | CTTCTGCTCCTTCAGTGTGAG |  |
| STAT3 | STAT3-qPCR-F | GTACCCAGACATCCCAAAAGAG |  |
|  | STAT3-qPCR-R | ATGGTATTGCTGAAGGAGGTG |  |
| STAT4 | STAT4-qPCR-F | TGGGTGGATCAGTTAGAAAATGG |  |
|  | STAT4-qPCR-R | TCAGAGGGTTTTCAGGAACG |  |
| STAT5B | STAT5B-qPCR-F | CAGTTACCTCATCTACGTGTTCC |  |
|  | STAT5B-qPCR-R | CTCACAAACTCTGGCACAAC |  |
| STAT6 | STAT6-qPCR-F | CAACCTCTACCCCAACACC |  |
|  | STAT6-qPCR-R | GTTTGTCCCTTTCGCTTTCC |  |
| β-actin | β-actin-qPCR-F | ATCTTTCTTGGGTATGGAGTC |  |
|  | β-actin-qPCR-R | GCCAGGGTACATTGTGG |  |
| Note. The underlined letters showed enzyme loci, the letters in front of them showed the protected base pairs, and the red letters showed the mutation sites. | | | |
